# A Narrative Review of the Co-Occurrence and Interaction of Childhood Emotional Neglect and Overprotection in Developmental Pathways to Disordered Eating

**DOI:** 10.3390/children12121643

**Published:** 2025-12-03

**Authors:** Valentina Lucia La Rosa, Federica Tortorella, Elena Commodari

**Affiliations:** Department of Educational Sciences, University of Catania, Via Biblioteca 4, 95124 Catania, Italy; federica.tortorella@studium.unict.it (F.T.); elena.commodari@unict.it (E.C.)

**Keywords:** developmental psychology, emotional neglect, parental overprotection, disordered eating, attachment, emotion regulation

## Abstract

**Highlights:**

**What are the main findings?**

- Though different in form, emotional neglect and parental overprotection converge as complementary relational risk factors that shape developmental pathways to disordered eating.
- Integrating attachment-based and schema-focused perspectives clarifies the ways in which early relational experiences influence emotion regulation, self-concept, and coping strategies associated with maladaptive eating patterns.

**What is the implication of the main finding?**

- Recognizing emotional neglect and overprotection as interconnected caregiving patterns underscores the importance of evaluating emotional deprivation and autonomy restriction in clinical and preventive settings.
- Combining emotion-focused work with autonomy-supportive approaches, such as family-based and attachment-informed strategies, may improve emotional clarity, strengthen agency, and reduce vulnerability to disordered eating.

**Abstract:**

Childhood emotional neglect and parental overprotection are two subtle yet influential caregiving patterns that shape emotional, relational, and self-regulatory development. Though they are often examined separately, emerging research suggests that they may act independently and together to increase vulnerability to disordered eating. This narrative review synthesizes contemporary theoretical and empirical contributions to clarify how emotional neglect and overprotection affect pathways related to emotion regulation, attachment processes, and self-concept development. The review introduces the concept of complementarity, explaining how these patterns may co-occur within the same family system or fluctuate among individual caregivers. This creates developmental contexts marked by emotional deprivation and restricted autonomy. Based on this synthesis, the manuscript presents an integrative framework that considers distinct and shared mechanisms connecting caregiving experiences to maladaptive eating patterns. Developmental and clinical implications are discussed, emphasizing the importance of assessing relational histories, supporting emotional clarity, and promoting autonomy in intervention planning. The limitations of the narrative approach and directions for future research are outlined, including the need for longitudinal designs, more precise measurement of caregiving dimensions, and a more systematic investigation of the interactive effects of emotional neglect and overprotection.

## 1. Introduction

Eating disorders are multifactorial conditions shaped by the interaction of biological, psychological, and interpersonal processes [1,2,3]. Alongside these clinical syndromes, a broader spectrum of maladaptive eating patterns, referred to as *disordered eating*, includes emotional eating, restrained eating, binge tendencies, and reliance on food for affect regulation [4,5,6]. These behaviors may emerge along a continuum of severity and do not necessarily reach diagnostic thresholds, yet they often reflect underlying vulnerabilities that can also contribute to the development or maintenance of eating disorders [7,8]. Understanding the developmental origins of such vulnerabilities requires attention on early relational experiences that shape emotion regulation, interpersonal functioning, and the construction of the self [9,10].

A growing body of evidence highlights the significance of early caregiving environments in shaping emotional and relational capacities associated with both disordered eating and increased risk for eating disorders [9,10,11,12]. Subtle forms of relational adversity that do not involve overt abuse may exert long-lasting effects on regulatory processes, autonomy development, and self-organization, domains closely implicated in eating-related difficulties [9,10,13].

Within this context, two caregiving dimensions emerge as particularly salient: *emotional neglect* and *parental overprotection*. Emotional neglect refers to the persistent absence of emotional availability, warmth, and responsiveness to the child’s affective needs [14,15,16]. Such environments impair the development of foundational skills in emotional awareness, self-soothing, and interpersonal security, contributing to vulnerabilities commonly observed in individuals who present with disordered eating patterns, including emotion regulation difficulties, attachment insecurity, and negative self-schemas [10].

Parental overprotection, by contrast, is characterized by excessive involvement, heightened parental anxiety, and restrictions on opportunities for independent exploration and decision-making [17]. Unlike coercive or authoritarian control, overprotection primarily reflects limited autonomy support and intrusive caregiving driven by fear, vigilance, and low parental confidence in the child’s competence [17]. Research indicates that overprotection is associated with reduced autonomy, external locus of control, dependence on others for regulation, and elevated anxiety, as well as heightened body-related concerns and dysregulated eating patterns [18,19].

Despite consistent evidence linking emotional neglect and overprotection to eating-related vulnerabilities, the literature lacks an integrative framework explaining how these dimensions may operate independently and jointly across development. Emotional neglect and overprotection are often conceptualized as opposing constructs, which may obscure the conditions under which they co-occur within the same family system and interact to shape psychological functioning [20,21]. Recent conceptual and empirical work suggests that these patterns may intersect through inter-parental asymmetry, intra-parental inconsistency, or complementary dynamics grounded in family anxiety, emotional inhibition, or dysregulated caregiving [17,22].

The present manuscript addresses this gap by offering a comprehensive theoretical examination of emotional neglect and parental overprotection and proposing an integrative framework connecting these caregiving dimensions to disordered eating. While the primary focus is on maladaptive eating patterns, we also acknowledge that the mechanisms discussed may contribute to vulnerability for eating disorders. We first review the theoretical foundations and empirical findings linking emotional neglect and overprotection to emotional and behavioral vulnerabilities relevant to eating behavior. We then introduce the concept of complementarity to clarify how these caregiving patterns may co-occur and interact. Building on this foundation, we elaborate the psychological mechanisms through which emotional neglect and overprotection may shape pathways toward disordered eating, focusing on emotion regulation, attachment processes, and self-concept development. Finally, we outline the clinical implications of this framework and offer directions for future research.

## 2. Methodology

This review adopts a narrative approach, which is well suited for synthesizing heterogeneous studies from developmental psychology, clinical science, attachment theory, and eating disorder research. Narrative reviews allow for theoretically informed integration of empirical findings, conceptual models, and clinical observations without imposing formal inclusion or exclusion criteria [23,24].

In line with methodological guidance for narrative reviews, the search process followed an iterative and flexible approach aimed at identifying conceptually relevant literature rather than applying predefined inclusion or exclusion criteria or database-specific search strings [24]. Exploratory searches were conducted across PsycINFO, PubMed, Scopus, and Google Scholar using keywords related to emotional neglect, parental overprotection, autonomy restriction, attachment processes, emotion regulation, self-concept, and disordered eating. Backward and forward citation tracking was used to identify additional relevant works. Particular emphasis was placed on research published from 2020 to 2025.

Study selection was guided by conceptual relevance rather than systematic criteria. Sources were included when they offered insights into caregiving patterns, developmental mechanisms, or eating-related outcomes. While not exhaustive, this approach allows for a comprehensive developmental synthesis grounded in contemporary evidence.

## 3. Results

### 3.1. Emotional Neglect and Associated Factors Linked to Disordered Eating

Emotional neglect has a distinctive place within the spectrum of early relational adversities because it unfolds quietly, often invisibly [16,25]. It refers to the persistent absence of emotional availability, warmth, and resonance in the caregiver–child relationship, even in the presence of adequate physical care [14,16]. Children exposed to this form of relational deprivation grow up in environments where their internal states are rarely mirrored or validated, and where emotional needs are neither explicitly rejected nor adequately met [26]. Precisely because of its subtlety, emotional neglect frequently remains unacknowledged within families, yet its developmental consequences are deep and long-lasting [14,15].

From a developmental perspective, one of the earliest and most pervasive effects of emotional neglect concerns the maturation of emotion regulation [27]. When caregivers do not notice or respond to a child’s affective signals, opportunities for co-regulation diminish, and the child receives limited scaffolding to recognize, organize, or soothe emotional states [10,28]. Across time, these early omissions shape difficulties both in accessing one’s emotions and in managing them adaptively [28,29,30]. Such patterns are particularly relevant for disordered eating, as difficulties in regulating distress and identifying internal cues frequently translate into reliance on food to modulate emotions, fluctuations between overcontrol and loss of control, or oscillations between avoidance and impulsivity in eating behavior [31,32].

Emotional neglect also interferes with the formation of secure attachment bonds [33]. Limited emotional responsiveness compromises the sense of being held in mind by caregivers, which is central to the development of internal security and relational confidence [30,34]. Insecure attachment, especially in its avoidant or disorganized forms, has been consistently associated with a range of emotional and interpersonal difficulties, including heightened anxiety, impaired emotion regulation, reduced social support, and fragile self-representations [35,36]. Therefore, it is consistently associated with self-reliance strategies that minimize emotional expression, reduced help-seeking, and heightened vulnerability to interpersonal stressors. These relational patterns often intersect with eating-related difficulties: individuals may turn to food as a stable and controllable source of comfort, or may experience eating and body-related concerns as domains less risky than close relationships, where emotional engagement feels unsafe [37].

A further consequence of emotional neglect concerns the development of the self. Recent work has shown that emotional neglect is associated with disturbances in the structural integration of personality, including low self-worth, internal fragmentation, and difficulties maintaining coherent self-representations [14]. These vulnerabilities are closely linked to core features of disordered eating and eating disorders, such as body dissatisfaction, internalization of appearance-related standards, and the use of eating behaviors to manage feelings of inadequacy or compensate for a fragile sense of self [38,39,40].

Taken together, these mechanisms illustrate why emotional neglect has emerged as a significant relational antecedent of disordered eating [41]. Empirical studies consistently show that individuals exposed to emotional neglect display higher levels of emotional eating, binge tendencies, rigid dietary restraint, and sensitivity to body-related feedback [9,10]. Emotional neglect is also linked to broader emotional and interpersonal difficulties, including anxiety, depression, and interpersonal insecurity, which frequently co-occur with disordered eating and intensify its course [11].

These converging lines of evidence suggest that the impact of emotional neglect on eating-related vulnerabilities is neither linear nor isolated. Instead, it shapes a developmental context marked by compromised regulation, fragile relational expectations, and a sense of self that oscillates between invisibility and inadequacy. Within such landscapes, disordered eating can emerge not as a sudden deviation, but as a strategy that fills gaps left by early relational omissions.

### 3.2. Overprotection and Associated Factors Linking to Disordered Eating

Parental overprotection is a caregiving pattern that has drawn increasing attention in developmental research because of its subtle but pervasive influence on children’s autonomy, perceived competence, and emotional adjustment [42]. Unlike coercive or authoritarian control, overprotection is not defined by overt demands or punitive strategies [43]. Rather, it reflects an anxious, intrusive, and excessively vigilant style of caregiving that restricts the child’s opportunities for exploration and independent problem solving [17]. In overprotective environments, children often grow up in a relational climate where the world is implicitly framed as dangerous, their abilities as insufficient, and unfamiliar situations as overwhelmingly risky [44]. As a result, they receive the message that autonomy is precarious and best avoided [42].

From a developmental perspective, the central issue in overprotection lies in the absence of autonomy support [45]. When caregivers intervene prematurely, anticipate difficulties, or manage challenges on behalf of the child, they inadvertently limit the development of self-efficacy, initiative, and flexible regulation strategies [17,42,46]. These experiences shape an internal landscape in which decisions feel daunting, mistakes feel threatening, and the self appears fragile or dependent [42,45]. Research consistently shows that overprotection is associated with heightened anxiety, fear of failure, and reliance on external sources of control, all of which have implications for eating-related vulnerabilities [42,46].

The mechanisms through which overprotection contributes to disordered eating reflect these developmental pressures. First, autonomy restriction may increase the likelihood that individuals turn toward eating as a domain where control feels tangible and accessible [47]. For some, food becomes a space in which emotional tension can be managed or displaced when direct action in other life areas feels overwhelming [48]. Second, overprotective caregiving is strongly linked to dependence and enmeshment schemas, in which closeness is maintained through intrusion rather than mutual responsiveness [17]. These schemas often manifest in heightened sensitivity to interpersonal expectations, body-related evaluation, and fear of negative judgment, which are well-established correlates of disordered eating [49].

Moreover, the anxious climate characteristic of overprotection can shape emotion regulation in developmentally specific ways. Instead of fostering experimentation and tolerance for uncertainty, overprotective parents tend to model worry, avoidance, and hypervigilance [42,45]. Children exposed to this style may learn to manage internal states by minimizing risk, seeking reassurance, or adopting rigid behavioral strategies [42,46]. Over time, these tendencies become fertile ground for eating behaviors that provide predictable and controllable routines, whether through strict food rules or through cycles of avoidance and overeating when distress becomes difficult to manage [18].

Recent evidence further illustrates how overprotection interacts with body-related processes. Individuals who report higher levels of parental overprotection also tend to exhibit greater body dissatisfaction, interpersonal insecurity, and drive for thinness, even in non-clinical samples [18,47]. These associations suggest that overprotection may increase the salience of external evaluation and heighten concerns about adequacy and appearance. The emphasis on safety and protection within the family can unintentionally reinforce vigilance toward potential threats in the social environment, including perceived scrutiny related to the body and weight.

Importantly, overprotection does not act in isolation. Its developmental consequences intersect with emotional, relational, and cognitive dynamics that can predispose individuals to disordered eating [18,47]. Autonomy restriction, heightened anxiety, and dependence coexist with a fragile or externally anchored sense of self, creating a psychological milieu where eating and body-related behaviors can offer a sense of structure or regulation [47,48]. Thus, while overprotection may initially appear benign compared to more overtly maladaptive forms of caregiving, its long-term implications for self-regulation and vulnerability to disordered eating are substantial and deserve careful theoretical and clinical attention.

### 3.3. Interaction/Complementarity Between Emotional Neglect and Overprotection

Although emotional neglect and parental overprotection are often conceptualized as distinct and, at times, opposing caregiving patterns, developmental research suggests that they may co-occur within the same family system and have complementary effects on children’s emotional, relational, and self-regulatory development [17,50]. Understanding their interaction is crucial for clarifying how subtle relational adversities may converge to shape vulnerabilities associated with disordered eating.

A first layer of complementarity emerges at the *inter-parental level*. In many families, caregiving responsibilities are neither evenly distributed nor characterized by the same emotional tone [51]. It is not uncommon for one caregiver to be highly anxious, intrusive, or overinvolved, while the other adopts a more emotionally withdrawn or disengaged stance [51,52]. This asymmetry may reflect differences in parental temperament, mental health, or stress exposure, creating an environment in which children oscillate between the hypervigilant protection of one caregiver and the muted emotional presence of the other [51]. Recent research on parental bonding and early maladaptive schemas supports this pattern, showing that individuals often report mixed caregiving profiles that combine elements of enmeshment with experiences of emotional deprivation [17]. Such inter-parental complementarity can amplify uncertainty about relational expectations and undermine the development of a coherent sense of safety.

A second form of complementarity can arise *within a single caregiver*. Parents navigating chronic stress, emotional dysregulation, or unresolved attachment issues may alternate between withdrawal and intrusive involvement depending on context, emotional state, or perceived threat [53]. For example, a caregiver who feels overwhelmed may become emotionally unavailable, yet shift into overprotective or controlling behavior when the child’s autonomy activates their anxieties about risk or vulnerability. These oscillations, although subtle, can be confusing for the child, who must adapt to inconsistent relational cues and navigate shifting expectations about closeness, independence, and emotional expression. This pattern is consistent with work linking parental emotional dysregulation to inconsistent caregiving strategies, as well as with evidence that both emotional neglect and overprotection emerge more frequently in families marked by high stress, parental anxiety, or ambivalent relational dynamics [10,22].

A third, more conceptual level of complementarity concerns the *psychological functions* that emotional neglect and overprotection may serve within families. Emotional neglect deprives the child of emotional resonance, leaving significant gaps in co-regulation, validation, and relational attunement. Overprotection, although qualitatively different, may operate as a compensatory strategy in which parents attempt to maintain closeness or manage their own anxieties about the child’s competence or well-being. When these dynamics coexist, children may receive minimal support for emotional exploration while simultaneously being discouraged from developing autonomy. In this sense, emotional neglect and overprotection converge to create an environment that undermines both emotional self-knowledge and independent action. The combination of emotional undernourishment and autonomy restriction can shape developmental trajectories characterized by fragile self-concept, heightened anxiety, and limited regulatory flexibility [54].

These forms of complementarity have specific implications for disordered eating. The interplay of emotional neglect and overprotection may create a developmental climate in which emotions feel unmanageable, relational expectations are unpredictable, and personal agency appears both risky and out of reach. In this context, eating-related behaviors can become a means to regulate distress, exercise a sense of control, or compensate for internal fragmentation. Research linking parental overprotection to body dissatisfaction, drive for thinness, and interpersonal insecurity [18,55], and emotional neglect to emotion regulation difficulties and self-concept disturbances [10], suggests that their combined influence may heighten vulnerability by shaping complementary, rather than redundant, pathways.

The potential interaction between emotional neglect and overprotection reflects the complex, multilayered nature of family systems. Rather than conceptualizing these patterns as mutually exclusive, recognizing their possible co-occurrence allows for a more comprehensive understanding of how relational environments can shape the emotional and cognitive architecture associated with disordered eating. This perspective provides an important stepping stone for articulating a comprehensive developmental framework capable of capturing the subtle yet powerful ways in which caregiving patterns intersect over time.

### 3.4. Associated Factors of Emotional Neglect, Overprotection and Disordered Eating

The psychological consequences of emotional neglect and parental overprotection do not unfold in isolation. Rather, they converge on a set of interconnected developmental processes that shape how individuals experience emotions, relate to others, and construct a sense of self. These processes offer an overarching framework for understanding why emotional neglect and overprotection, despite their differences, may both contribute to vulnerability for disordered eating.

A first shared domain concerns *emotion regulation*. The pathways are distinct yet complementary. Emotional neglect limits the child’s access to co-regulatory experiences and emotional scaffolding, resulting in difficulties identifying and modulating internal states [14,54]. Overprotection, by contrast, restricts opportunities for independent emotional problem solving. When caregivers intervene prematurely or communicate excessive worry, children receive fewer chances to tolerate uncertainty, recover from distress, or practice coping strategies autonomously [42]. Across development, these trajectories may converge in adolescents and adults who rely on eating behaviors to manage emotional arousal, reduce anxiety, or avoid internal states that feel overwhelming [10,18,48].

The second domain is *attachment*. Emotional neglect disrupts the formation of secure internal working models by limiting the caregiver’s availability as a consistent source of comfort and attunement [56]. This is often reflected in avoidant or disorganized attachment patterns, characterized by inhibited emotional expression, relational distance, and difficulties seeking support [29]. Overprotection, conversely, is frequently associated with anxious-ambivalent dynamics in which closeness is maintained through intrusion rather than mutual responsiveness [17,29]. Although the attachment consequences differ, both patterns generate relational environments in which emotional needs feel risky, unclear, or inadequately met. This relational insecurity is linked to disordered eating in multiple ways, including the use of food as a safe substitute for interpersonal soothing, heightened sensitivity to relational evaluation, and an overreliance on external rules to manage self-worth [55,57,58].

A third shared domain involves the *development of the self*. Emotional neglect is strongly associated with deficits in self-coherence, low self-worth, and internal fragmentation, as children who grow up with little emotional resonance may struggle to develop a stable sense of who they are [16,33]. Overprotection, on the other hand, is associated with dependence, perceived incompetence, and reduced confidence in one’s ability to act autonomously [42]. These trajectories differ in their origins but converge in their effects: a fragile, externally anchored sense of self that is highly sensitive to external feedback, including feedback related to the body [10,19,47]. Such vulnerabilities can heighten susceptibility to body dissatisfaction, internalization of appearance ideals, and reliance on eating-related behaviors as a way to construct or stabilize self-evaluations [55].

Importantly, these domains do not act independently. Emotion regulation difficulties often amplify attachment insecurity, while insecure attachment shapes the development of self-concept, and a fragile self makes emotional experiences more difficult to manage [59]. In this recursive cycle, disordered eating may become a strategy for restoring a sense of order, predictability, or control. The empirical literature reinforces this integrative view: studies have shown that individuals exposed to either emotional neglect or overprotection report heightened anxiety, interpersonal insecurity, and greater reliance on eating-related coping strategies [10,18]. These findings underscore the importance of considering not only the distinct contributions of emotional neglect and overprotection, but also the shared developmental mechanisms through which they exert risk.

Taken together, these associated factors illustrate how emotional neglect and overprotection can shape a developmental landscape that is simultaneously marked by emotional vulnerability, relational uncertainty, and a fragile or externally referenced sense of self. In such contexts, disordered eating may offer temporary relief or a compensatory sense of control, even as it reinforces the underlying vulnerabilities that gave rise to it. These converging mechanisms set the stage for the integrative framework presented in the following section.

## 4. Discussion

### 4.1. Proposed Framework

The developmental patterns associated with emotional neglect and parental overprotection converge on a set of vulnerabilities that can shape eating-related behaviors across infancy, childhood, adolescence, and young adulthood [9,10,18]. The goal of the proposed framework is to integrate these pathways into a coherent model that explains how relational environments marked by emotional unavailability, autonomy restriction, or their co-occurrence may increase susceptibility to disordered eating. This framework is based on well-established developmental theories and recent empirical studies showing the psychological consequences of emotional neglect and overprotection [9,10,17,18].

One assumption is that emotional neglect, overprotection, and their potential interaction influence disordered eating through a few core psychological mechanisms: emotion regulation, attachment processes, and self-concept development. These mechanisms form a developmental architecture through which caregiving experiences shape vulnerability. Importantly, this framework conceptualizes these domains as interconnected rather than independent, reflecting a relational-developmental perspective [10,18].

A second assumption is that emotional neglect and overprotection generate distinct, yet partially overlapping, vulnerabilities. Emotional neglect primarily disrupts emotional resonance, which limits the development of emotional awareness, co-regulation, and secure internal working models [54]. Overprotection, on the other hand, restricts autonomy and fosters dependence, conveying implicit messages that the world is dangerous, that mistakes are threatening, and that the child’s competencies are insufficient [17,42]. Recent studies show that children exposed to overprotection often exhibit anxious vigilance, an external locus of control, and reliance on others for reassurance [42,46]. When emotional neglect and overprotection co-occur, these vulnerabilities may intersect, creating profiles marked by both emotional undernourishment and restricted agency [22].

A third assumption is that eating behaviors may serve regulatory, compensatory, or identity-related functions in individuals whose developmental contexts involve emotional neglect, overprotection, or both. Studies show that emotional eating, binge tendencies, and strict dietary restraint are frequently associated with emotion regulation difficulties, insecure attachment, and fragile self-representations [10,12,48]. In the absence of reliable internal cues or a stable relational base, eating and body-related behaviors may offer predictable strategies for modulating emotions, reducing anxiety, or restoring a sense of control [48].

Building on these assumptions, the proposed framework outlines three principal pathways connecting caregiving patterns to disordered eating.

*Pathway 1: Emotional Neglect→Emotional and Self-Development Vulnerabilities→Disordered Eating*

Emotional neglect limits emotional co-regulation and validation, hindering the development of adaptive emotion regulation, secure attachment, and coherent self-representations [10,16]. These difficulties can promote reliance on food for soothing, avoidance of internal states, and alternating restrictive or impulsive eating patterns. Studies consistently link emotional neglect to emotional eating, binge episodes, and heightened body dissatisfaction [9].

*Pathway 2: Overprotection→Autonomy and Competence Vulnerabilities→Disordered Eating*

Overprotection conveys messages of fragility and danger, undermining autonomy, fostering dependence, and heightening anxiety [17,42,46]. These developmental pressures are associated with body dissatisfaction, drive for thinness, and interpersonal insecurity [18]. Individuals exposed to overprotection may use eating and body-related behaviors as a way to regain control, manage uncertainty, or stabilize self-worth in contexts where autonomy feels unsafe [19,47].

*Pathway 3: Complementarity of Emotional Neglect and Overprotection→Combined Vulnerabilities→Disordered Eating*

When emotional neglect and overprotection co-occur (either across caregivers or within the same caregiver over time), they may amplify one another’s effects [22]. Emotional deprivation limits internal security, while autonomy restriction limits agency. This combination produces a developmental environment marked by unmanageable emotions, relational inconsistency, and a fragile or externally anchored sense of self. In these contexts, disordered eating may emerge as a compelling strategy to regulate distress, create structure, or build a sense of coherence [10,49,60].

The framework’s distinctive contribution is its ability to explain the heterogeneity of eating-related vulnerabilities. Rather than attributing risk to one caregiving dimension, it integrates emotional neglect, overprotection, and their interaction into a developmental model that accounts for both emotional and autonomy-related mechanisms. This approach aligns with contemporary calls for relational-developmental models of disordered eating and eating disorders [3,61] and provides a foundation for targeted assessment and intervention, which are addressed in the subsequent section.

### 4.2. Clinical Implications

Understanding how emotional neglect and parental overprotection shape developmental vulnerabilities offers a number of meaningful implications for the assessment and treatment of eating disorders. The framework proposed in this review highlights the importance of attending to subtle relational adversities that may not emerge in traditional screening but nonetheless exert profound effects on emotional functioning, interpersonal patterns, and self-concept. Integrating these dynamics into clinical work allows for a more precise formulation of the mechanisms maintaining disordered eating and opens new avenues for targeted intervention.

A first implication concerns *assessment*. Emotional neglect and overprotection are rarely assessed systematically in clinical settings, yet their developmental consequences closely mirror the emotional and relational difficulties commonly observed in individuals with eating disorders [15,62]. Including measures of emotional responsiveness, autonomy support, and parental intrusiveness can help clinicians identify histories of relational deprivation or overinvolvement that contribute to emotion regulation problems, interpersonal sensitivity, or fragile self-worth. Instruments such as the Parental Bonding Instrument [63] or schema-based assessments can provide an initial indication of whether early caregiving environments were characterized by emotional omission, autonomy restriction, or a combination of both [49]. Importantly, these assessments become relevant to eating disorders precisely because the mechanisms implicated in emotional neglect and overprotection closely map onto processes that sustain emotional eating, restrictive tendencies, and body-related distress.

A second implication relates to *case formulation*. A developmental formulation that incorporates emotional neglect and overprotection helps clinicians trace the specific pathways through which patients may have learned to rely on eating behaviors for regulation or identity-related functions. For individuals with a history of emotional neglect, interventions may need to prioritize the development of emotional awareness and the capacity to recognize, tolerate, and express internal states [64]. Patients whose histories reflect overprotection may benefit from work that targets autonomy, decision-making, and the internalization of self-efficacy [17]. When emotional neglect and overprotection co-occur, clinicians may encounter patients who struggle with both unregulated emotional experiences and difficulties asserting agency, a combination that can intensify reliance on eating behaviors [10,18,65]. Formulating these patterns explicitly offers a more detailed understanding of the patient’s relationship with food, beyond symptom-based descriptions.

Third, the framework suggests specific *directions for intervention*. Emotion-focused and skills-based approaches may be particularly effective for individuals with histories of emotional neglect, as they address deficits in emotional clarity and regulation [66,67]. For those shaped by overprotection, interventions that support autonomy, reduce fear-based avoidance, and strengthen internal decision-making may foster greater flexibility [68]. In cases where these dimensions intersect, treatment may require careful balancing of emotional exploration with autonomy-promoting strategies, ensuring that patients develop both regulatory capacities and a sense of agency. Family-based interventions may also benefit from recognizing these dynamics, especially when parents continue to exhibit anxious or withdrawn relational patterns that reinforce the patient’s vulnerabilities [54,69].

Finally, recognizing the clinical significance of complementarity between emotional neglect and overprotection has several implications for treatment planning. Patients who have experienced both insufficient emotional attunement and restricted autonomy may present with complex relational expectations, alternating between emotional withdrawal and dependence. These patterns can manifest in the therapeutic relationship and require clinicians to navigate moments of avoidance, reassurance-seeking, or heightened sensitivity to interpersonal cues [70,71,72]. Addressing these dynamics directly, and linking them to early relational experiences, can help patients develop more coherent internal models and reduce reliance on disordered eating as a compensatory strategy.

### 4.3. Future Directions

The framework presented in this manuscript highlights several avenues for future research that can meaningfully advance our understanding of how emotional neglect and parental overprotection contribute to the development of disordered eating. Although the literature has begun to connect these caregiving patterns to eating-related vulnerabilities, much remains to be clarified regarding their developmental timing, mechanisms of action, and interactive effects. Addressing these gaps will require coordinated methodological and theoretical innovation.

A first priority concerns the need for longitudinal studies capable of tracing how emotional neglect and parental overprotection shape psychological processes across development. Existing evidence is largely cross-sectional or retrospective, making it difficult to determine whether emotion regulation difficulties, attachment insecurity, or fragile self-concept function as mechanisms that precede the emergence of disordered eating or as consequences of eating-related distress [10,18]. Prospective studies beginning in early childhood and extending into adolescence and emerging adulthood would allow researchers to examine how caregiving patterns interact with temperament, peer relationships, or identity development to shape eating-related trajectories.

A second direction involves refining the measurement of emotional neglect and overprotection. Many commonly used instruments capture broad parenting dimensions but may not adequately distinguish between emotional unavailability and autonomy restriction. Recent work on early maladaptive schemas, parental emotional socialization, and parental anxiety offers promising pathways for developing multidimensional, developmentally sensitive measures [17]. Multi-method approaches combining self-report, observational paradigms, and ecological momentary assessment could provide a more nuanced understanding of caregiving dynamics as they unfold in naturalistic contexts.

Future research should also explore the interaction between emotional neglect and overprotection in more depth. The framework presented here proposes several pathways through which these patterns can co-occur, yet empirical studies explicitly examining their joint influence are scarce. Designs that assess both caregiving dimensions simultaneously, and that test their combined effects on psychological mediators, could clarify whether emotional neglect and overprotection represent additive, synergistic, or distinct risk factors. This line of inquiry is especially relevant given emerging evidence that emotional neglect and overprotection may interact to shape complex profiles of emotional and autonomy-related vulnerabilities [18,22,63].

Another important direction involves examining how specific eating-related outcomes relate to emotional neglect and overprotection. Although the umbrella term “disordered eating” is useful for capturing a range of maladaptive behaviors, future research would benefit from differentiating between emotional eating, restrained eating, binge tendencies, purging behaviors, and body dissatisfaction. Distinct patterns may be more strongly linked to emotional neglect (for example, emotion-driven eating and difficulties interpreting internal cues) or to overprotection (such as concerns about evaluation, perfectionistic tendencies, or rigid control strategies).

Further, there is a need to investigate developmental moderators that may influence the pathways from caregiving to eating behaviors. These include child temperament, gender-related socialization, peer experiences, and social media exposure [73,74,75,76]. Such moderators may either buffer or intensify the influence of caregiving patterns on eating-related vulnerabilities, and their examination could help identify subgroups of adolescents or young adults who are particularly susceptible.

Finally, the framework has important implications for intervention research. Future studies could test whether treatments that specifically target emotion regulation difficulties, attachment processes, or autonomy development led to greater improvements in eating symptoms among individuals with histories of emotional neglect, overprotection, or both. Integrating family dynamics into treatment for adolescents and young adults, particularly when parental anxiety or emotional unavailability remain active contributors, represents another promising direction.

### 4.4. Limitations

This narrative review presents a theoretically informed synthesis of the literature on emotional neglect and parental overprotection, yet several methodological limitations should be acknowledged. First, narrative reviews are not designed to achieve replicability or exhaustiveness. Although we conducted extensive searches across major databases and integrated a broad range of recent empirical and theoretical work, the selection of studies was guided by conceptual relevance rather than by predefined inclusion or exclusion criteria. As such, the review may not capture all available evidence, and future systematic reviews or meta-analyses could complement our approach by providing quantitative estimates of associations between caregiving patterns and eating-related outcomes.

Second, much of the empirical evidence on emotional neglect, parental overprotection, and their developmental consequences is derived from retrospective self-report designs [18,47]. These methods are susceptible to recall bias, which is the systematic distortion that occurs when individuals report on past events or experiences. This distortion often alters the perceived timing, duration, or emotional significance of these events due to reconstructive memory processes or current psychological states [77,78]. This limitation reduces the accuracy with which early caregiving experiences can be recalled. Therefore, prospective longitudinal studies are needed to clarify when and how specific caregiving patterns begin to influence emotion regulation, attachment processes, or self-concept development.

Third, the constructs examined are defined and measured inconsistently across studies. Emotional neglect and parental overprotection overlap conceptually with related dimensions such as parental psychological control, emotional availability, or inconsistent caregiving, making it difficult to compare findings directly. Greater conceptual clarity and measurement refinement would enhance the precision of future investigations.

Finally, the developmental mechanisms discussed in this review are not specific to disordered eating. Emotional neglect and overprotection may contribute to a wide range of internalizing, interpersonal, and self-regulatory difficulties, and only a subset of individuals exposed to these caregiving patterns will develop clinically significant eating disorders. Future research should examine how these trajectories diverge across different domains of functioning and identify the moderators that channel early caregiving into distinct developmental outcomes.

Despite these limitations, this review provides a conceptually grounded, updated, and integrative framework for understanding how subtle caregiving patterns may shape vulnerability to disordered eating across development.

## 5. Conclusions

Emotional neglect and parental overprotection represent two subtle yet powerful dimensions of early caregiving that shape children’s emotional, relational, and self-regulatory development in ways that may contribute to vulnerability for disordered eating. Although these patterns have often been studied separately, the evidence reviewed in this manuscript underscores the importance of examining their distinct and shared developmental trajectories, as well as the conditions under which they may co-occur. By integrating these insights into a developmental framework, we highlight how relational environments marked by emotional unavailability, restricted autonomy, or their interaction may influence emotion regulation, attachment processes, and the construction of the self, ultimately shaping eating-related behaviors across adolescence and young adulthood.

The proposed framework contributes to the field by offering a more nuanced understanding of how caregiving patterns intersect to influence vulnerability for disordered eating. Rather than interpreting emotional neglect and overprotection as isolated risk factors, the model situates them within a broader relational-developmental architecture that emphasizes how early relational experiences can shape regulatory and identity-related processes. This perspective aligns with the growing recognition that eating disorders emerge from complex, multidetermined trajectories and that their developmental antecedents must be understood within the child’s broader relational ecology.

Clinically, this framework underscores the importance of assessing early caregiving experiences and attending to how emotional neglect, autonomy restriction, or their combination may continue to shape patients’ emotional functioning and relational expectations. Recognizing these dynamics can enrich case formulations, guide intervention targets, and help clinicians tailor treatment to the patient’s developmental history. From a research perspective, the framework highlights several avenues for future investigation, including longitudinal studies, refined measurement approaches, and more integrated examinations of how emotional neglect and overprotection jointly influence psychological mechanisms central to eating-related vulnerability.

Ultimately, this manuscript argues for a relational-developmental approach to disordered eating and eating disorders, one that acknowledges the enduring influence of early caregiving while emphasizing the psychological processes through which these influences unfold. By strengthening conceptual clarity and integrating empirical findings across the literature, the framework aims to support both scientific understanding and clinical practice, offering a foundation for more precise, developmentally informed models of disordered eating.

## Data Availability

No new data were created or analyzed in this study. Data sharing is not applicable to this article.
